# SH3YL1 Protein Predicts Renal Outcomes in Patients with Type 2 Diabetes

**DOI:** 10.3390/life13040963

**Published:** 2023-04-07

**Authors:** Sang Youb Han, Seung Hyun Han, Jung Yeon Ghee, Jin Joo Cha, Young Sun Kang, Dae Ryong Cha

**Affiliations:** 1 Department of Internal Medicine, Inje University, Ilsan-Paik Hospital, Goyang 10380, Republic of Korea; 2 Department of Internal Medicine, Korea University, Ansan Hospital, Ansan 15368, Republic of Korea

**Keywords:** SH3YL1, diabetes, kidney, NOX4, biomarker

## Abstract

NADPH oxidase (NOX)-derived oxidative stress is an important factor in renal progression, with NOX4 being the predominant NOX in the kidney. Recently, Src homology 3 (SH3) domain-containing YSC84-like 1 (SH3YL1) was reported to be a regulator of NOX4. In this study, we tested whether the SH3YL1 protein could predict 3-year renal outcomes in patients with type 2 diabetes. A total of 131 patients with type 2 diabetes were enrolled in this study. Renal events were defined as a 15% decline in the estimated glomerular filtration rate (eGFR) from the baseline, the initiation of renal replacement therapy, or death during the 3 years. The levels of the urinary SH3YL1-to-creatinine ratio (USCR) were significantly different among the five stages of chronic kidney disease (CKD) and the three groups, based on albuminuria levels. The USCR levels showed a significant negative correlation with eGFR and a positive correlation with the urinary albumin-to-creatinine ratio (UACR). Plasma SH3YL1 levels were significantly correlated with UACR. The highest tertile group of USCR and plasma SH3YL1 had a significantly lower probability of renal event-free survival. Furthermore, the highest tertile group of USCR showed a significant association with the incidence of renal events after full adjustment: adjusted hazard ratio (4.636: 95% confidence interval, 1.416–15.181, *p* = 0.011). This study suggests that SH3YL1 is a new diagnostic biomarker for renal outcomes in patients with type 2 diabetes.

## 1. Introduction

Diabetic kidney disease (DKD) is the leading cause of end-stage kidney disease worldwide, with a prevalence of approximately 50%. Several mechanisms have been found to contribute to the development and progression of DKD [1], with oxidative stress being one of the key factors. Oxidative stress induces lipid peroxidation, DNA damage, and protein nitration, which augment other inflammatory changes, such as the recruitment of inflammatory cells and the synthesis of reactive oxygen species (ROS) [2,3]. ROS act as secondary signal mediators where the elevation of their levels induces glomerular hypertrophy, tubulointerstitial injury and matrix protein accumulation in various kidney diseases, including DKD [3].

DKD is one of the serious microvascular complications in patients with diabetes, and the diagnosis of DKD is made based on the status of albuminuria and the estimated glomerular filtration rate (eGFR). However, many patients with albuminuria do not progress to end-stage renal disease, and many patients with chronic kidney disease with decreased eGFR do not have albuminuria. Therefore, many clinical and experimental studies have been performed to identify novel biomarkers that could improve the diagnostic accuracy in earlier stages of DKD. Kidney-specific biomarkers provide information on renal or systemic changes in various renal regions, such as the glomerulus or tubules [4,5], pathological changes in the extracellular matrix (ECM) metabolism, and on inflammation, endothelial dysfunction, oxidative stress and fibrosis [6,7,8]. However, there are no established biomarkers for clinical application, and the development of new biomarkers is urgently needed to better understand the cellular and molecular mechanisms of DKD. As one of the most important pathogenetic mechanisms, oxidative stress has been considered to be a leading cause of diabetic microvascular complications, including diabetic nephropathy [9,10]. In diabetic conditions, renal oxidative stress is increased due to many pathological stimuli, such as chronic hyperglycemia, alongside many diabetes-associated stimuli, including advanced glycation end product, angiotensin II, TGF-b, and dyslipidemia [11,12,13].

Nicotinamide adenine dinucleotide phosphate (NADPH) oxidases (NOXs) are a major endogenous source of cellular ROS [14]. Their role includes maintaining homeostatic functions such as phagocytosis in normal circumstances; however, pathological NOX activation in infection and the diabetic milieu induces an increased synthesis of ROS [15]. Several regulatory molecules tightly regulate ROS generation to prevent cytotoxicity [16]. NOX4, one of the seven known NOX family members, is the predominant NOX in the kidney, and a major source of ROS in DKD [17,18].

Several regulators of NOX4 have been reported in the literature, including P22phox, gp91phox, and P47phox [15,19,20]. Recently, Src homology 3 (SH3) domain-containing YSC84-like 1 (SH3YL1) was reported to be a regulator of NOX4 [21]. SH3YL1 is a highly conserved noncatalytic protein in yeasts and vertebrates that binds to cytoplasmic tyrosine kinase [22,23]. It regulates the hair cycle and hair follicle formation in the keratinocytes of the skin and is expressed in various human organs, including the kidney [22,24].

Recently, we found that the SH3YL1 protein is produced from renal cells, such as podocytes, mesangial cells, and proximal tubule cells, in response to high glucose stimulation, and is associated with oxidative stress-induced inflammatory processes in the diabetic condition. These results imply a possible new physiological role for SH3YL1 in renal injury in diabetes. Although the origin of plasma SH3YL1 is not clear, we recently observed that aortic endothelial cells secrete SH3YL1 in response to LPS stimulation [25]. In addition, we found that peritoneal mesothelial cells can secrete large amounts of SH3YL1 in continuous peritoneal dialysis patients with peritonitis [25].

We also reported that plasma SH3YL1 protein is a novel biomarker for type 2 DKD in animals and humans [25]. However, due to the limitation of this cross-sectional study, the predictive role of this protein could not be proven. Here, we tested whether the SH3YL1 protein could predict 3-year renal outcomes in patients with type 2 diabetes.

## 2. Materials and Methods

### 2.1. Study Patients

A total of 131 patients with type 2 diabetes from two different hospitals were enrolled in this study. Patients with type 2 diabetes were included regardless of their renal function, except for those undergoing renal replacement therapy. The major exclusion criteria were systemic diseases that required corticosteroids, hepatic diseases, and malignancies. Baseline blood and urine samples were collected, centrifuged at 3000× *g* for 10 min, and stored at −80 °C until analysis.

### 2.2. Renal Outcome

To determine renal outcomes, we defined renal events as a 15% decline in eGFR from the baseline, the initiation of renal replacement therapy, or death during the 3 years. In total, 116 out of 131 patients were analyzed to predict renal outcomes after excluding 15 patients with an eGFR < 15 mL/min/1.73 m^2^. Of the 116 patients, laboratory data were available at 3 years for 99 patients who were analyzed for the prediction of renal outcomes.

### 2.3. Data Collection

The data collected in the study included the patients’ demographics and comorbidities at 1 year prospectively, and at 2 and 3 years retrospectively. In addition, body mass index (BMI) was calculated as weight(kg)/height²(m²), and both systolic and diastolic blood pressure (SBP and DPB, respectively) were recorded. Baseline laboratory examinations included serum creatinine, blood urea nitrogen (BUN), fasting blood glucose, HbA1c, hemoglobin, white blood cell count, platelets, total protein, albumin, uric acid, total cholesterol, low-density lipoprotein cholesterol (LDL), high-density lipoprotein cholesterol (HDL), triglycerides, and urinary albumin and creatinine levels. Plasma and urinary creatinine levels were measured using a modified Jaffe method. eGFR was calculated using the Chronic Kidney Disease Epidemiology Collaboration (CKD-EPI) equation: eGFR = 141 × min (SCr/κ, 1)^α^ × max (SCr/κ,1)^−1.209^ × 0.993^Age^ × 1.018 (if female) × 1.159 (if black), where κ is 0.7 for females and 0.9 for males, and α is −0.329 for females and −0.411 for males, respectively.

### 2.4. Measurement of Plasma SH3YL1 and Urinary Albumin and SH3YL1

Urinary albumin excretion was measured using an immunoturbidimetric assay (Roche Diagnostics, Mannheim, Germany). Plasma levels of SH3YL1 were measured using an enzyme-linked immunosorbent assay (MyBioSource, Inc., San Diego, CA, USA). Urinary levels of albumin and SH3YL1 were corrected using the urinary creatinine concentration: urinary albumin-to-creatinine ratio (UACR) and urinary SH3YL1-to-creatinine ratio (USCR).

### 2.5. Statistical Analysis

Statistical analysis was performed using SPSS version 25 (IBM Corp, Armonk, NY, USA). ANOVA or the Kruskal–Wallis test was used to compare statistical differences in the USCR and plasma SH3YL1 levels among the CKD stages and UACR groups. The Chi-square test was performed for categorical data. The correlation between plasma or urine SH3YL1 levels and biochemical parameters was analyzed using Spearman’s rank test. Kaplan–Meier curves were analyzed to estimate the probability of renal event-free survival over time according to the tertiles of USCR or plasma SH3YL1 levels. Cox proportional hazards survival regression analyses were used to investigate the effect of USCR or plasma SH3YL1 levels with several variables related to renal survival for up to 3 years. The effects are expressed as hazard ratios (HR) with 95% confidence intervals (CIs). Adjustments for the clinical and biological parameters were performed by including these parameters as covariates in the regression models. All data are presented as mean ± standard deviation (SD) or as a median and interquartile range (IQR). A *p*-value < 0.05 was regarded as significant.

## 3. Results

### 3.1. Clinical Characteristics of the Study Population

The mean age of the 131 patients (89 men and 42 women) was 62.7 ± 11.4 years, and the mean time since diagnosis with diabetes was 13.1 ± 8.3 years P(Table 1). The median values of eGFR and UACR were 46.0 mL/min/1.73 m^2^ (28.8–78.3) and 0.173 g/gCr (IQR 0.016–1.000), respectively. The median value of USCR was 7.73 pg/mgCr (IQR 2.84–25.5), and that of plasma SH3YL1 was 301.1 pg/mL (IQR 154.3–1035.8). Other baseline characteristics are shown in Table 1.

Baseline characteristics were classified into three groups based on tertiles of USCR or plasma SH3YL1 levels (Table 1). The tertiles based on the USCR showed significant differences in age, eGFR and ACR. Several factors were significantly different among the tertiles of plasma SY3YL1 levels, including hemoglobin, BUN, uric acid, protein, albumin, and UACR.

### 3.2. Plasma and Urinary Levels of SH3YL1

**Figure 1:**
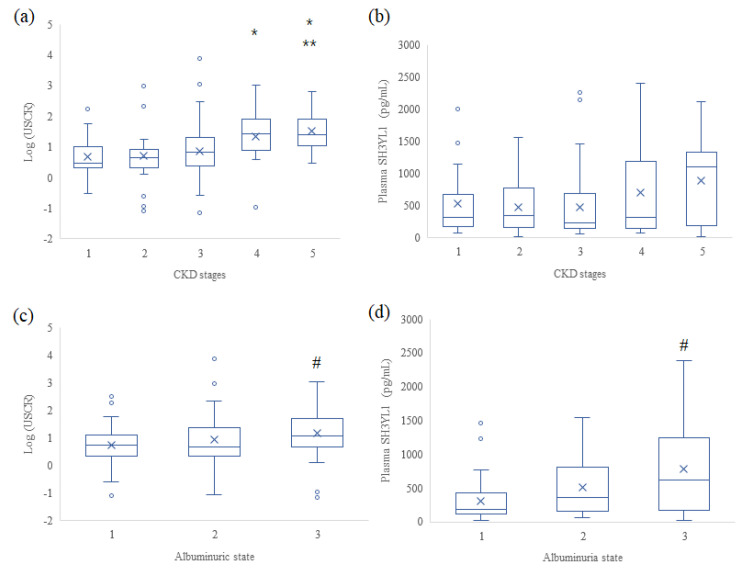
Urinary and plasma SH3YL1 levels according to the CKD stages (**a**,**b**) and albuminuric state (**c**,**d**) in patients with diabetes. Albuminuric states were classified into normoalbuminuric microalbuminuric and macroalbuminuric states. The urinary SH3YL1-to-creatine ratio (USCR, pg/mgCr) was log-transformed. All data are presented as the median and interquartile range. * *p* < 0.05 vs. CKD stage1 and 2, ** *p* < 0.05 vs. CKD stage 3, # *p* < 0.05 vs. microalbuminuric state; Kruskal–Wallis test.

The USCR was significantly different among the 5 stages of CKD according to the non-parametric analysis (Figure 1). The USCR levels in CKD stages 4 and 5 were significantly higher than those in CKD stages 1 and 2. In addition, the levels in CKD5 were significantly higher than those in CKD stage 3. However, plasma SH3YL1 levels did not differ among the CKD groups.

The USCR and plasma SH3YL1 levels were also different among the three UACR groups according to the Kruskal–Wallis test (Figure 1). The USCR was significantly higher in the macroalbuminuric group than in the normoalbuminuric group. Plasma SH3YL1 levels also showed similar patterns to those of the USCR in the three UACR groups.

**Figure 2:**
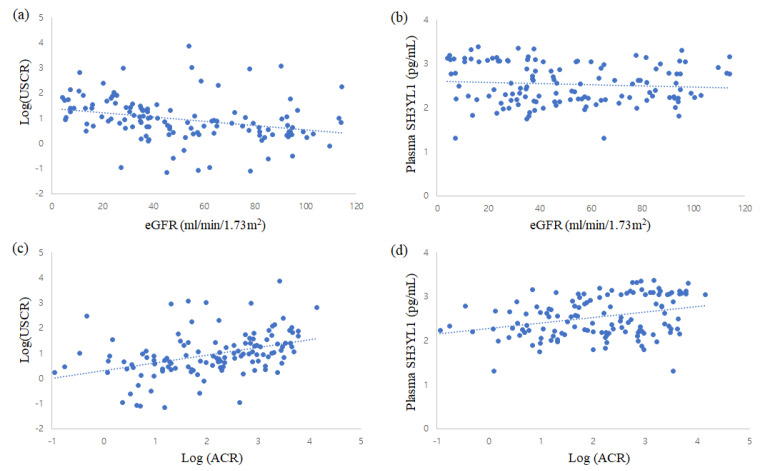
Correlation between SH3YL1 and eGFR (**a**,**b**) and albuminuria (**c**,**d**). The urinary SH3YL1-to-creatine ratio (USCR, pg/mgCr) and urinary albuminuria (ACR, mg/gCr) were log-transformed.

Correlations between SH3YL1 and other clinical parameters were analyzed using Spearman’s rank test. There was a significant negative correlation between the USCR and eGFR (r = −0.445, *p* < 0.001), and a positive correlation between the USCR and UACR (r = 0.488, *p* < 0.001) (Figure 2). In addition, the levels were significantly correlated with SBP (r = 0.223, *p* = 0.013), hemoglobin (r = −0.433, *p* < 0.001), and total protein (r = −0.348, *p* < 0.001). Plasma SH3YL levels were significantly correlated with the UACR (r = 0.302, *p* < 0.001), but not with eGFR. The plasma SH3YL1 levels were also significantly correlated with age (r = −0.311, *p* < 0.001), total protein (r = −0.178, *p* = 0.049), and LDL (r = 0.206, *p* = 0.030).

### 3.3. Prediction for Renal Outcome

**Figure 3:**
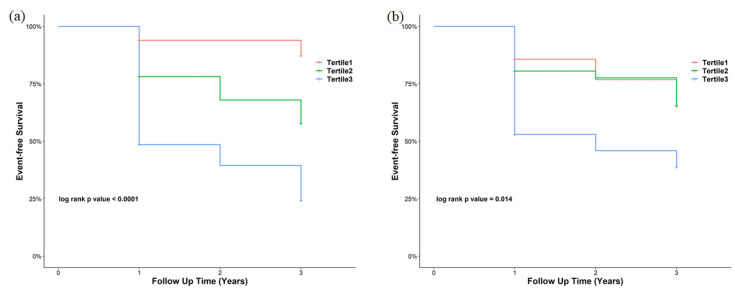
Kaplan–Meier curves for renal outcomes by tertiles based on (**a**) the urinary SH3YL1-to-creatinine ratio and (**b**) plasma SH3YL1 levels.

Kaplan–Meier curves showed the rate of disease-free survival in the three tertiles of the USCR and plasma SH3YL1 levels (Figure 3). The highest tertile group of the USCR and plasma SH3YL1 had a significantly higher probability of representing renal outcome. To confirm whether urinary and plasma SH3YL1 levels could accurately predict the renal outcome, a Cox proportional hazard regression was used. Patients were classified into three groups based on the tertiles of the USCR and plasma SH3YL1 levels in order to remove the strong skewness effect in the levels. The tertiles of the USCR and plasma SH3YL1 levels were analyzed using several clinically important factors in different models. The USCR was significantly predictive in the unadjusted model. This significance also persisted in the sex- and age-adjusted model. In the fully adjusted model with sex, age, SBP, BMI, HbA1c, eGFR and UACR, the highest-tertile group showed a significant association with the incidence of renal events (adjusted HR, 4.636; 95% CI, 1.416–15.181; *p* = 0.011) (Table 2). The plasma SH3YL1 level was also a significant predictive factor in the unadjusted model. However, it was not significant in the sex- and age-adjusted or fully adjusted models (Table 2).

## 4. Discussion

This study presents the clinical significance of SH3YL1 as a diagnostic biomarker of renal outcomes in patients with type 2 diabetes. The USCR was found to be significantly correlated with eGFR and the UACR. Plasma SH3YL1 levels were also correlated with the UACR. The highest tertile group of the USCR and plasma SH3YL1 had a significantly lower probability of renal event-free survival. Furthermore, the highest tertile group of the USCR could predict 3-year renal outcomes.

To the best of our knowledge, only two reports on SH3YL1 in kidney disease models have been published. Recent reports have shown that SH3YL1 is a regulator of NOX4 [21] and is a biomarker in animal models and patients with diabetes [25]. NOX4 is a well-known contributor to acute and chronic kidney injury, including cisplatin-induced injury, unilateral ureteral obstruction and DKDs [26,27,28]. Yoo et al. reported that SH3YL1 interacts with the Nox4-p22^phox^ complex, stimulating H_2_O_2_ production. LPS-induced SH3YL1 knock-out mice exhibited less inflammatory cell infiltration and mild renal dysfunction compared to LPS-injected wild-type mice. These results suggest that SH3YL1 contributes to stabilizing the NADPH binding site of NOX4 [21]. This suggests that SH3YL1 plays a role in NOX4-induced kidney injury, and is a NOX-related candidate marker in disease models.

DKD is characterized by a thickening of the glomerular basement membrane (GBM) and mesangial expansion, and a histologically increased accumulation of ECM proteins. Among many cells in the glomeruli, podocytes have been considered to play a pivotal role in the development and progression of DKD. Renal injury in patients with diabetes is reflected by increased urinary albumin excretion and decreased GFR. According to the progression of renal dysfunction, many pathological changes occur in the kidney, such as mesangial expansion, podocyte apoptosis and tubulointerstitial fibrosis [29,30]. The progression of DKD is characterized by glomerular hypertrophy and the inflammation of glomeruli, and fibrosis is accompanied by the accumulation of ECM in the tubulointerstitial tissues. Although multiple complicated mechanisms are involved in the progression of DKD, oxidative stress is closely associated with renal inflammation and fibrosis [3,31,32].

ROS are constitutively synthesized in all tissues and play an important role as mediators of cell signaling in numerous physiologic processes, including cell proliferation, death, differentiation and immune defense [33]. However, if the balance between the synthesis and removal of ROS is disrupted, oxidative stress is induced, leading to organ injury. Excessive ROS production has been reported in many kidney diseases, including hypertensive nephropathy diabetic nephropathy, acute kidney injury and immune-mediated glomerulonephritis [31]. NOXs catalyze the transfer of electrons from NADPH to molecular oxygen, and are known to be major sources of ROS production in the kidney [34]. There are seven NOX isoforms (Nox1–5, Duox1, Duox2); among these, Nox1, Nox2, Nox4 and Nox5 are widespread and are expressed throughout the renal tissues and cells [35]. Among the seven Nox isoforms, Nox4 is the most abundant isoform in the kidney and has been reported to play a central role in type 1 and 2 diabetes-induced oxidative stress. Accordingly, many experimental studies targeting Nox4 have been performed, and several studies have focused on Nox4 as a potential therapeutic target for DKD [9,10]. A high glucose condition increases Nox4 expression in cultured mesangial cells, and an increased Nox4-dependent ROS concentration induces glomerular expansion and mesangial ECM accumulation. Furthermore, oxidative stress and mesangial ECM accumulation are attenuated in streptozotocin-induced *Nox4* knock-out and *ApoE/Nox4* double knock-out mice [9,36]. A previous study demonstrated that the administration of antisense oligonucleotides for Nox4 in type 1 diabetic rats improved renal hypertrophy and matrix expansion [37]. Collectively, these results suggest that Nox4 plays a critical role in the pathophysiology of DKD. Recently, Yoo et al. reported that SH3YL1 is a cytosolic regulatory protein for Nox4 lipopolysaccharide (LPS)-induced H_2_O_2_ generation, and is involved in an animal model of LPS-induced acute kidney injury [21].

Our previous data also showed that SH3YL1 is a promising marker in a diabetic kidney injury model [15]. SH3YL1 expression was increased in both podocytes and proximal tubule cells stimulated by high glucose and angiotensin II. The intrarenal expression of SH3YL1 was evidently upregulated in db/db mice. Immunoreactivity was observed in the glomeruli and tubulointerstitial areas, especially in podocytes and proximal tubule cells. Interestingly, chronological changes were observed in db/db mice, while no changes were shown in db/m mice. Furthermore, plasma SH3YL1 levels were also increased in patients with type 2 diabetes, with a significant trend according to the albuminuric state. The current study examined whether SH3YL1 was a predictive marker in patients with type 2 diabetes, showing that the USCR was a significant marker for predicting 3-year renal outcomes. Considering the link between SH3YL1 and NOX4, in addition to detecting SH3YL1 in various tissues, including the kidney, SH3YL1 might be a potential biomarker for the reflection of NOX4-related oxidative stress in DKD.

The classical biomarkers for DKD are microalbuminuria, decreased creatinine clearance, and an increased serum creatinine level [38]. However, decreased renal function is not always accompanied by an increase in urinary albumin excretion. It has been reported that around 20–30% of DKD patients with decreased renal function show normal albuminuria [39]. In the early stages of DKD, urinary albumin excretion and eGFR are usually well-maintained; however, molecular changes induced by diabetes, such as inflammation and oxidative stress, are progressively activated. Therefore, the diagnostic tests routinely used in clinical practice, including serum creatinine, eGFR, and albuminuria, are limited in their capacity to detect DKD early. Thus, there is a need for the discovery of new and noninvasive biomarkers for DKD. Promising new biomarkers in DKD are urinary neutrophil gelatinase-associated lipocalin, kidney injury molecule-1, serum cystatin C, uromodulin, urinary N-acetylo-beta-D-glucosaminidase, the liver-type fatty acid binding protein, and serum interleukin 18 [40]. These new biomarkers are associated with glomerular dysfunction, tubular dysfunction, oxidative stress, and inflammation [41]. Although oxidative stress has been considered to have a central pathogenic role in DKD, there are only a few oxidative stress-related biomarkers in DKD. 8-hydroxy-2′-deoxyguanosine (8-OHdG)—a product of oxidative DNA damage that is circulated in the plasma and excreted in the urine—is proposed as a promising biomarker for DKD [42]. A higher plasma concentration of 8-OHdG has been reported as an independent risk factor for kidney disease in patients with type 1 diabetes, suggesting that 8-OHdG is a possible biomarker for predicting the progression of DKD [42]. However, in patients with type 2 diabetes and healthy controls, the urinary 8-OHdG level was inferior to the urinary albumin excretion when predicting the development of DKD [43].

In this study, the plasma SH3YL1 level was correlated with the UACR according to the non-parametric analysis, in accordance with previous results [25]. In addition, the highest patient tertile demonstrated worse outcomes in the survival curve. The SH3YL1 levels were significantly predictive of the 3-year renal outcome in the unadjusted and sex- and age-adjusted models, but not in the fully adjusted model. Plasma levels should be further confirmed as a predictive marker in future studies using large numbers of patients.

Recent reports have revealed the various roles of SH3YL1 in kidney function; these include its interaction with the androgen receptor [44], its association with the immunological non-responsiveness of HIV-infected patients [45], its role in influencing T-cell activation [46], and its involvement in the membrane invagination process of endosomes [47]. Currently, the role of SH3YL1 in various diseases is unclear. Considering the link between SH3YL1 and NOX4, it is necessary to confirm the significance of this biomarker in kidney diseases, in addition to detecting its level in various tissues, including the kidney.

Our study had several limitations. First, this study did not include control groups, such as a healthy population or non-diabetic CKD patients. Second, a small number of patients were enrolled, and the outcome data for 14.6% of the 116 patients were missing due to a lack of follow-up. Third, men were enrolled more than women, which might have led to selection bias. Fourth, the distribution of urinary and plasma SH3YL1 levels was positively skewed. A non-parametric analysis was performed to address this issue. Fifth, we did not measure the SH3YL1 levels during the follow-up period. This would show a clear association between SH3YL1 and other clinical parameters. Sixth, we did not measure oxidative stress-related markers. Although they are known to be associated with SH3YL1 levels, it is still unclear which markers are reliable. Candidate markers should be clarified in future studies.

## 5. Conclusions

Plasma and urinary SH3YL1 levels were found to be significant predictive markers of renal outcomes. These results support our previous findings, demonstrating that plasma SH3YL1 is a promising new biomarker for diabetic nephropathy. Despite its limitations, this study showed clinical significance in patients with type 2 diabetes. Future longitudinal studies with a large number of patients are necessary to further elucidate the clinical significance of SH3YL1.

## Figures and Tables

**Table 1:** Baseline characteristics in all patients, and three groups based on tertiles of the USCR and plasma SH3YL1 in patients with an eGFR ≥ 15 mL/min/1.73 m^2^.

Characteristics	Total		USCR				Plasma SH3YL1		
		Tertile 1	Tertile 2	Tertile 3	*p*-Value	Tertile 1	Tertile 2	Tertile 3	*p*-Value
Number (M:F)	89:42	29:7	31:5	22:15	0.021	30:8	27:11	28:10	0.723
Age (years)	62.7 ± 11.4	66.3 ± 9.3	66.0 ± 10.2	57.3 ± 11.8	<0.001	62.2 ± 9.6	62.8 ± 12.0	64.4 ± 11.6	0.685
BMI	25.4 ± 3.51	25.5 ± 2.10	25.5 ± 3.50	24.7 ± 4.09	0.503	24.5 ± 3.18	25.5 ± 3.68	25.7 ± 3.01	0.274
Duration (y)	13.1 ± 8.3	12.2 ± 8.1	13.6 ± 8.5	12.1 ± 7.7	0.660	11.4 ± 7.8	12.9 ± 8.5	13.4 ± 8.3	0.567
SBP (mmHg)	135.8 ± 14.8	133.5 ± 14.2	134.5 ± 11.2	136.7 ± 18.5	0.633	132.1 ± 11.8	135.4 ± 14.6	137.2 ± 18.1	0.345
DBP (mmHg)	74.1 ± 11.9	72.1 ± 12.6	72.3 ± 11.6	77.0 ± 11.4	0.131	73.4 ± 10.9	75.3 ± 12.8	71.4 ± 11.4	0.357
FBS (mg/dL)	131 (105.7–154.5)	128.5 (114.0–149.3)	129.0 (105.0–164.0)	131.0 (109.0–152.0)	0.967	118.0 (92.0–148.0)	138.5 (120.3–158.5)	130.0 (114.5–173.3)	0.054
HbA1c (%)	6.90 (6.40–8.05)	6.75 (6.40–7.43)	6.90 (6.50–8.05)	7.00 (6.40–8.20)	0.463	6.90 (6.60–7.40)	6.95 (6.40–7.50)	7.30 (6.43–9.20)	0.521
BUN (mg/dL)	30.0 ± 20.1	26.3 ± 12.0	26.5 ± 21.1	24.9 ± 13.5	0.892	20.7 ± 19.8	26.3 ± 12.6	30.5 ± 12.6	0.029
Creatinine (mg/dL)	1.95 ± 1.61	1.48 ± 0.60	1.38 ± 0.54	1.64 ± 0.85	0.244	1.15 ± 0.38	1.59 ± 0.69	1.77 ± 0.77	<0.001
eGFR (ml/min/1.73 m^2^)	46.0 (28.8–78.3)	78.0 (51.0–92.8)	46.4 (35.1–71.9)	35.6 (28.0–56.4)	<0.001	53.6 (35.9–64.5)	53.1 (35.7–81.2)	50.9 (32.5–89.6)	0.903
Hemoglobin (g/dL)	12.58 ± 2.26	13.1 ± 2.13	13.4 ± 1.87	12.5 ± 1.89	0.163	13.9 ± 1.89	13.0 ± 2.10	12.1 ± 1.66	0.001
Total protein (g/L)	6.94 ± 0.60	7.07 ± 0.47	6.88 ± 0.45	6.85 ± 0.83	0.238	7.09 ± 0.58	7.02 ± 0.52	6.72 ± 0.59	0.018
Albumin (g/L)	4.12 ± 0.49	4.19 ± 0.44	4.18 ± 0.38	3.99 ± 0.62	0.154	4.24 ± 0.50	4.21 ± 0.39	3.94 ± 0.48	0.011
Cholesterol (mg/dL)	152.0 (132.0–183.5)	152.9 (136.2–182.3)	138.0 (129.7–161.0)	155.5 (138.6–192.8)	0.096	143.3 (131.0–163.1)	153.7 (135.9–180.9)	155.2 (132.3–191.3)	0.380
HDL (mg/dL)	46.0 ± 11.7	43.1 ± 5.37	49.1 ± 14.4	45.8 ± 13.2	0.569	52.4 ± 17.0	38.9 ± 7.96	46.9 ± 10.0	0.082
LDL (mg/dL)	89.9 ± 30.8	83.2 ± 23.7	87.5 ± 23.3	99.0 ± 41.6	0.082	90.9 ± 28.0	83.4 ± 23.3	94.4 ± 38.9	0.327
Triglyceride (mg/dL)	132.0 (97.0–200.0)	126.0 (90.0–200.0)	127.0 (97.0–190.0)	153.0 (105.5–209.5)	0.704	145.0 (100.3–218.8)	111.0 (90.3–181.5)	139.5 (104.0–191.0)	0.287
Uric acid (mg/dL)	6.62 ± 2.10	6.91 ± 2.13	6.39 ± 1.98	6.56 ± 2.11	0.558	5.80 ± 1.80	6.84 ± 1.94	7.23 ± 2.29	0.012
UACR (g/gCr)	0.173 (0.016–1.000)	0.019 (0.005–0.099)	0.165 (0.014–0.562)	0.720 (0.052–1.652)	<0.001	0.088 (0.010–0.627)	0.045 (0.010–0.338)	0.285 (0.053–1.778)	0.011
USCR (pg/mgCr)	7.73 (2.84–25.5)	1.92 (0.72–2.48)	6.60 (4.79–8.33)	34.1 (20.4–104.3)	<0.001	8.97 (2.65–34.1)	4.52 (1.92–11.0)	7.53 (3.10–21.6)	0.098
Plasma SH3YL1 (pg/mL)	301.1 (154.3–1035.8)	242.9 (163.5–585.1)	433.1 (172.4–1119.9)	181.2 (141.5–647.4)	0.194	129.7 (96.5–151.6)	265.6 (206.2–403.2)	1133.1 (771.9–1318.5)	<0.001

Data are expressed as numbers (%) and means ± SD or median (IQR). Abbreviations: UACR, urinary albumin-to-creatinine ratio; USCR, urinary SH3YL1-to-creatinine ratio; BMI, body mass index; SBP, systolic blood pressure; DBP, diastolic blood pressure; FBS, fasting blood glucose; eGFR, estimated glomerular filtration rate; HDL, high density lipoprotein; LDL, low density lipoprotein; BUN, blood urea nitrogen.

**Table 2:** Unadjusted and adjusted HRs for the risk of renal events according to SH3YL1 levels.

Markers	Number of Events/Number of Patients	Unadjusted		Model 1		Model 2	
		HR (95% CI)	*p*-Value	aHR (95% CI)	*p*-Value	aHR (95% CI)	*p*-Value
Urine SH3YL1-to-Cr ratio							
Tertile 1	4/33	Ref.		Ref.		Ref.	
Tertile 2	13/32	3.778 (1.231–11.597)	0.020	3.772 (1.228–11.584)	0.020	2.633(0.809–8.576)	0.108
Tertile 3	26/35	8.326 (2.888–24.003)	<0.001	9.616 (3.291–28.099)	<0.001	4.636 (1.416–15.181)	0.011
Serum SH3YL1							
Tertile 1	12/35	Ref.		Ref.		Ref.	
Tertile 2	12/36	0.995 (0.447–2.215)	0.991	1.065 (0.475–2.390)	0.879	1.075 (0.473–2.441)	0.863
Tertile 3	20/34	2.167 (1.053–4.458)	0.036	2.448 (1.124–5.333)	0.024	1.511 (0.643–3.549)	0.343

Primary renal events are defined as a 15% decline in eGFR from the baseline. Model 1: adjusted for sex and age. Model 2: adjusted for body mass index, systolic blood pressure, HbA1c, eGFR, and urinary albumin-to-creatinine ratio in addition to model 1. Abbreviation: aHR, adjusted hazard ratio; CI, confidence interval; eGFR, estimated glomerular filtration rate.

## Data Availability

All data generated or analyzed during this study are included in this article. Further enquiries can be directed to the corresponding author.
